# Physical Layer Secret-Key Generation Scheme for Transportation Security Sensor Network

**DOI:** 10.3390/s17071524

**Published:** 2017-06-28

**Authors:** Bin Yang, Jianfeng Zhang

**Affiliations:** College of Information Engineering, Northwest A&F University, Xianyang 712100, China; b_yang@nwsuaf.edu.cn

**Keywords:** transportation security, secret-key generation, physical layer security, wireless sensor network

## Abstract

Wireless Sensor Networks (WSNs) are widely used in different disciplines, including transportation systems, agriculture field environment monitoring, healthcare systems, and industrial monitoring. The security challenge of the wireless communication link between sensor nodes is critical in WSNs. In this paper, we propose a new physical layer secret-key generation scheme for transportation security sensor network. The scheme is based on the cooperation of all the sensor nodes, thus avoiding the key distribution process, which increases the security of the system. Different passive and active attack models are analyzed in this paper. We also prove that when the cooperative node number is large enough, even when the eavesdropper is equipped with multiple antennas, the secret-key is still secure. Numerical results are performed to show the efficiency of the proposed scheme.

## 1. Introduction

Wireless sensors are widely used in transportation systems to ensure the system security, lower the fuel consumption and increase system efficiency etc. [1,2,3]. Cargo shipments security is a critical challenge for shippers, every year cargo theft costs billions of dollars. A Transportation Security Sensor Network (TSSN) architecture is developed to realize the vision of trusted corridors [3]. In TSSN the cargo security is monitored with active and battery-powered container seals (sensors) to report security seal events timely. Any unauthenticated attempts to unlock the container will be reported to the operations center through mobile network and internet. A security Seal Interrogation Transceiver (SIT) is designed to communicate with the container seals over a wireless network, which should be secured with secret-key, or else an adversary could interfere the wireless communication link to disable the alert system.

The security issue in TSSN is similar of that in Wireless Sensor Networks (WSNs). There are two main challenges on the secure communication: 1. the low cost of wireless nodes leads to severe resource constraints such as limited battery power, memory and low computation capability; 2. the open nature of the wireless link makes it easy to be eavesdropped. The normally used public cryptography approaches are not suitable for TSSN because sensor nodes (container seals) are source constrained devices that cannot afford for public key cryptography. Therefore, symmetric key-based schemes are widely used in WSNs, because of the advantages of low cost in power comsumption, time execution and code size [4,5]. The main challenge for WSNs to implement symmetric key-based scheme is the secret-key distribution in the network.

Physical layer key generation schemes could offer a solution of the issue [6]. The main advantage of physical layer key generation scheme is that the key is directly generated in physical layer, and there is not any key distribution process. In an ideal situation the eavesdropper could not get any information about the key.

The theoretical aspects of secrecy extraction from correlated random source have been firstly studied by [6,7]. It is shown that correlated observations of random sources could be used to distill secret-keys by discussing over a public channel, while the information rate leaked to the eavesdropper can be arbitrarily low. The supremum of achievable secret-key rate is called secret-key capacity. In recent years, significant interests in developing practical approaches to generate secret-key between multiple users have been attracted [8,9,10,11,12,13,14,15,16,17,18,19,20,21,22,23,24,25,26,27,28,29,30,31,32,33,34]. It is shown in [9,11] that there is a trade-off between the secret-key rate and the public communication rate in the key agreement protocols.

One of the main issues of these schemes is how to find proper random sources for secret-key generation. Such sources should create correlated randomness between the legitimate users, would have high level of randomness, and should be difficult for the eavesdropper to observe.

In this paper we propose a method to create artificial correlated random sources for wireless sensors to generate secret-key in a cooperative TSSN. The random sources are created by multiple nodes in the system, when the cooperative helpers send independent symbols simultaneously, different channel vectors result in different receiving signals, which prevents the eavesdropper from getting a copy of the legitimate users’ signals. Since the random source is artificially generated, even when CSI (Channel State Information) of the wireless channels changes slowly, high secret-key rate can still be achievable.

In the proposed scheme, the helpers have no idea of the receiving signals of the users. When the eavesdropper is equipped with multiple antennas or there are multiple eavesdroppers, it is possible for them to get what the helpers send. However, since the eavesdroppers have no idea about the legitimate nodes’ CSI, they still cannot get what the legitimate users get. It is proven that, from computational complexity security point of view, the proposed scheme is secure with enough helpers even when the antenna number of the eavesdropper is unlimited.

In [8], the random source is also artificial signals, secret-key is generated by opportunistic transmission over the quasi-static fading channel by sending signals when the channel condition of the legitimate users are better than the eavesdropper’s. However, this approach is based on certain assumptions that are hard to be realized in practice, while the proposal in this paper does not have. In [27,33], authors have investigated the impact of cooperative relay nodes on the secret-key generation, these algorithms are based on the wireless channel reciprocity of the users and relays. Since the achievable secret-key rate scales linearly with the number of relays, when there are large number of available relays, better system performance could be achieved. While the proposed scheme in this paper can generate secret-key artificially, our proposal is more suitable for the system with relatively small number of relays. The scheme of [34] is based on the knowledge of the eavesdropper and the communication capacity of the nodes is unlimited, that is not practical in a real system.

The organization of the paper is as follows. Section 1 introduces the proposed scheme of the system. Section 2 presents secret-key rate analysis. Section 3 studies the system security with different thread models. Numerical result is presented in Section 4. Finally, we conclude the paper in Section 5.

## 2. Proposed Scheme

**Figure 1:**
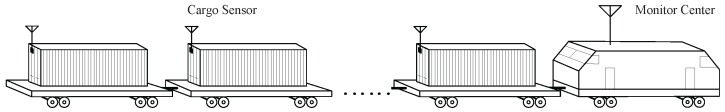
System Configure. In the system, cargo sensors (CS) are placed on the cargo containers to monitor or secure the containers. The monitor center (MC) is normally placed in locomotive. MC communicate with CS through wireless link, which should be secured by cryptography algorithm.

The system is shown in Figure 1, which is a wireless sensor network equipped in a rail-borne cargo. There are two types of nodes in the network. One is Cargo Sensor (CS), which is placed on the cargo container. Normally CS is battery-powered and designed to monitor security seal events or the status of the cargo. The other type of node in the network is Monitor Center (MC), which could be installed in the cab of the locomotive. CS reports sensor data to MC or receives commands from MC through secure wireless link, which is encrypted by symmetric cryptographic scheme. In this paper, we propose a novel key agreement or key distribution scheme in TSSN.

**Figure 2:**
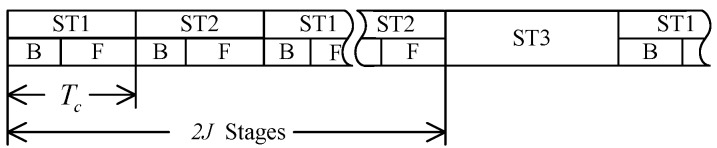
Communication frame structure.

**Figure 3:**
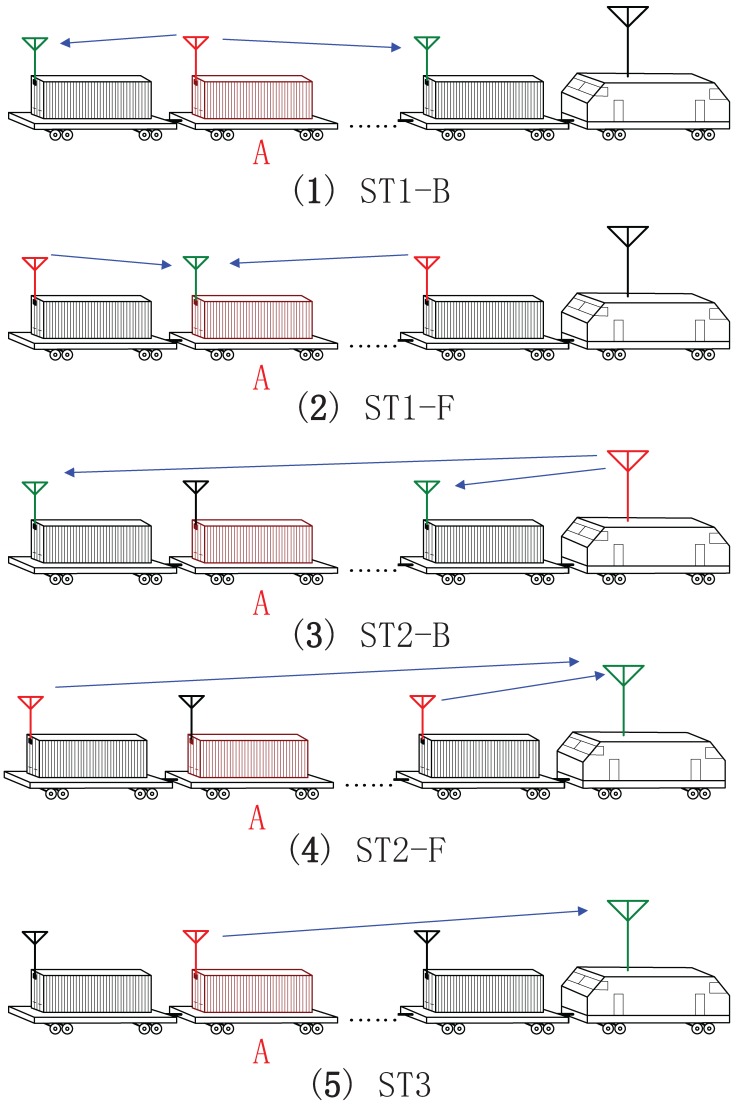
Algorithm steps. (**1**) A sends channel estimating sequence to helpers; (**2**) helpers transmit random signals simultaneously to A; (**3**) MC sends channel estimating sequence to helpers; (**4**) helpers send reversed random signals simultaneously to MC; (**5**) A communicates with MC to get agreement on the secret-key. Note that in the figure when sending signals the antenna is colored in red, when receiving signals the antenna is colored in green.

The proposed scheme is shown in Figure 2 and Figure 3. MC tries to update the secret-key of one of the CSs which is marked as A (the red container in Figure 3). All the communication parties can communicate with each other through a public channel. Other CSs can help MC and A to achieve the goal, we call them helpers. Note that helpers are not fixed, A could also play as a helper when MC wants to update the key of another CS.

Here we assume the number of the helpers is N,N≥3. A passive eavesdropper is located at somewhere trying to crack the secret-key, he can access the signals from all the communication partners. The system is a narrow band system, the wireless channels are assumed to be block fading with the coherent time $T_{c}$. Note that all the nodes in the system are equipped with single antenna.

The railway radio propagation environment is significantly impacted by the railway structures, such as cuttings, viaducts, and tunnels etc., so the wireless channel’s propagating characteristics changes while the train traveling from one site to another. Since the system is a narrow band system, the channel can be modeled as a complex random variable. The complex random channel gain is considered to keep constant within the coherent time $T_{c}$. Then the nodes in the system have to measure and renew the channel gain when the transmitting time is longer than $T_{c}$.

There are three stages in the proposed scheme.

In Stage 1 (ST1) within $T_{c}$, all the helpers synchronously send random symbols to A to create stochastic signals at A. There are two sub-stages in ST1: the first is the backward signal transmission sub-stage (ST1-B) from partner A to the helpers. In this sub-stage, User A sends channel estimation sequence to the helpers, then they can estimate the channel coefficients of the links from A to helpers. Due to the radio propagation reciprocity, the helpers then know the channel gains of the links from themselves to A. On the other hand, the eavesdropper can also get the channel estimation sequence from A, then he can estimate the channel gain between himself and A. Because user A and helpers do not send any messages about the channel information, the eavesdropper cannot get any information about the helpers’ links directly.

The second sub-stage in ST1 is forward signal transmission sub-stage (ST1-F) from the helpers to user A. All the helpers individually send *K* random symbols to user A, then the symbols that user A receives are

(1) $$x\left(k\right)=\sum_{i=1}^{N}h_{i}s_{i}\left(k\right)+n_{1}\left(k\right)$$

where $h_{i},\;i=1,\ldots ,N$ denotes the complex channel gains between the helpers and A, $s_{i}\left(k\right),\;$i=1,…,N denotes complex zero-mean Gaussian random symbols sent by the helpers which are independent of each other, and $n_{1}\left(k\right)$ denotes the receiving noise of user A. Here we assume that $|h_{i}{|}^{2}\neq 0$, which is a reasonable assumption for a practical system. Then the transmitting power of the helpers is $P_{i}=E(|s_{i}{|}^{2}),\;i=1,\ldots ,N$. In the proposed scheme, we set all the transmitting power to be the same, that is $P_{i}=P,\;i=1,\ldots ,N$.

During Stage 2 (ST2), all the helpers repeat the *K* symbols sent in the first stage multiplied with weight factors $w_{i},\;i=1,\ldots ,N$. There are also two sub-stages in this stage. The first is the backward sub-stage (ST2-B) for channel estimation. In this sub-stage, MC send channel estimation sequence to the helpers to estimate the complex channel gain. In addition, the eavesdropper also can only get the channel information between MC and himself. Next is the forward sub-stage (ST2-F) for the helpers to repeat the *K* symbols, then the symbols that MC receives are

(2) $$y\left(k\right)=\sum_{i=1}^{N}g_{i}w_{i}s_{i}\left(k\right)+n_{2}\left(k\right),$$

where $g_{i},\;i=1,\ldots ,N$ denotes the complex channel gains between the helpers and MC, and $n_{2}\left(k\right)$ is the noise of MC. We also assume that $|g_{i}{|}^{2}\neq 0$. To create correlated sources for the partners, we set

(3) $$w_{i}=\sqrt{\rho }\frac{h_{i}}{g_{i}},\;i=1,\ldots ,N,$$

where ρ is power factor to adjust the total transmitting power in the second stage. Note that $w_{i}$ is only determined by the helper’s own CSI and a global factor ρ, no other global information should be shared between the helpers. Then the receiving symbols of MC are

(4) $$y\left(k\right)=\sqrt{\rho }\sum_{i=1}^{N}h_{i}s_{i}\left(k\right)+n_{2}\left(k\right).$$

Then $y\left(k\right)=\sqrt{\rho }x\left(k\right)-\sqrt{\rho }n_{1}\left(k\right)+n_{2}\left(k\right)$, when signal power is high enough, the symbols received by MC and A are almost the same.

Note that if $|g_{i}|$ is a small low value, which means the link between MC and helper *i* is very weak. Then the transmit power of helper *i* during ST2-F has to be very high, and possibly exceeds the maximum transmit power of the helper. If the helper repeat the random symbols only with maximum power, MC’s receiving signals will not be the same as A’s without considering noises. There are three solutions: One is that MC can use antenna with high antenna gain, for example, using directional antenna, then usually $|g_{i}|$ will be lower $|h_{i}|$. The other solution is that helpers can adjust the global power factor ρ to make sure all the helper’s transmit power in ST2 is lower than the upper limit. However, this solution has the risk to leak some information of the legitimate user’s channel information. The last one is that the helper *i* is mute in ST2-F when the transmit power should be higher than the maximum power of himself. In an additional stage, helper *i* broadcast his signals sent in ST1, and tells every one that he is mute in ST2. Then A can remove what the helper *i* sends in his receiving signals with the check-sum information from MC in ST3. This additional stage will not influence the model and analysis in the paper.

In Stage 2, we assume perfect channel estimation of $h_{i}$ and $g_{i}$, which is not true in a practical system. When considering channel estimation errors, the receiving signals of MC will be

(5) $$\begin{array}{cc} y^{\prime }\left(k\right) & =\sqrt{\rho }\sum_{i=1}^{N}\frac{h_{i}+\Delta h_{i}}{g_{i}+\Delta g_{i}}g_{i}s_{i}\left(k\right)+n_{2}\left(k\right)\\ & =\sqrt{\rho }\epsilon \sum_{i=1}^{N}h_{i}s_{i}\left(k\right)+\sqrt{\rho }n_{\Delta }\left(k\right)+n_{2}\left(k\right), \end{array}$$

where $\Delta h_{i}$ and $\Delta g_{i}$ are the channel estimation errors, and

(6) $$\epsilon =\frac{\sum_{i=1}^{N}\frac{h_{i}+\Delta h_{i}}{g_{i}+\Delta g_{i}}g_{i}h_{i}^{*}}{\sum_{i=1}^{N}{\left|h_{i}\right|}^{2}},\;\;n_{\Delta }\left(k\right)=\sum_{i=1}^{N}\left[\frac{h_{i}+\Delta h_{i}}{g_{i}+\Delta g_{i}}g_{i}-\frac{\sum_{i=1}^{N}\frac{h_{i}+\Delta h_{i}}{g_{i}+\Delta g_{i}}g_{i}h_{i}^{*}}{\sum_{i=1}^{N}{\left|h_{i}\right|}^{2}}h_{i}\right]s_{i}\left(k\right).$$

It is easy to know that

(7) $$E_{s_{i}}\left(n_{\Delta }\left(k\right){\left(\sum_{i=1}^{N}h_{i}s_{i}\left(k\right)\right)}^{*}\right)=0,$$

which means $n_{\Delta }\left(k\right)$ can be considered as additional Gaussian noise. Then channel estimation errors will result in an additional noise to the receiving signals of MC, and in turn decrease the performance of the system. In this paper, we mainly focus on the upper bound of the system performance, the further discussion of the model in a real system is left for future work.

During the above two stages, the eavesdropper gets the signals as

(8) $$v_{1}\left(k\right)=\sum_{i=1}^{N}e_{i}s_{i}\left(k\right)+n_{E1}\left(k\right),\;v_{2}\left(k\right)=\sqrt{\rho }\sum_{i=1}^{N}e_{i}\frac{h_{i}}{g_{i}}s_{i}\left(k\right)+n_{E2}\left(k\right),$$

where $e_{i},\;i=1,\ldots ,N$ denotes the complex channel gains between the helpers and the eavesdropper, $n_{E1}\left(k\right)$ and $n_{E2}\left(k\right)$ are the noises of the eavesdropper in the two stages respectively.

Note that all the noise terms $n_{1}$, $n_{2}$, $n_{E1}$ and $n_{E2}$ are zero-mean white independent complex Gaussian random variables with the variance $\sigma_{n}^{2}$.

After 2J stages of transmission, there should be a stage of hand-shaking, which is Stage 3 (ST3). During ST3, A and MC exchange information to distill a common secret-key. In this paper, we concentrate on the class of key agreement protocols in which only A sends messages to MC. A computes a secret-key M and sending message C from *x*, then sends C to MC over the public channel, the total length of these sequences is JK. MC then computes the key $M^{\prime }$ from *y* and C. A secret-key rate $R_{k}$ is achievable if for any ϵ>0 and all sufficiently large number JK, there is a secret-key agreement such that

(9) $$\begin{array}{ccc} \frac{H(M)}{JK} & \geq & R_{k}-\epsilon , \end{array}$$

(10) $$\begin{array}{ccc} Pr\{M\neq M^{\prime }\} & \leq & \epsilon ,\;(\mathrm{Reliability}\;\mathrm{Condition}) \end{array}$$

(11) $$\begin{array}{ccc} \frac{I(M;v_{1},v_{2},C)}{JK} & \leq & \epsilon .\;(\mathrm{Sec}\mathrm{recy}\;\mathrm{Condition}) \end{array}$$

Then secret-key capacity is defined as the supremum of secret-key rates achievable for the model.

Since the length of C is relatively long, the transmission of C may be divided into several frames, which depends on the coherent time $T_{c}$. Here we do not show any details on ST3, for the information about the link between A and B is public, and does not affect the key generation process in this paper.

In general, a closed-form expression of the secret-key capacity is still an open problem. Nevertheless, in [6,7], upper bound and lower bound of the secret-key capacity are shown. Since we focus on the design of the correlated sources for secret-key generation in this paper, a tight upper bound is good enough for demonstrating the effectiveness of the proposal.

## 3. Secret-Key Rate Analysis

### Security Capacity of the Scheme

The model in this paper is a typical source model (Section 4.1 in [35]) of secret-key agreement, which represents a situation in which the two parties in the system observe the realizations of a random source to generate secret-key. A closed-form expression for the secret-key capacity for a general source model remains elusive. Then from the results in [6,7,35], with considering the one-way public communication between the legitimate users, the secret-key rate of our model is bounded as

(12) $$I\left(x;y\right)-I\left(x;v\right)\leq C_{sk}\leq I\left(x;y|v\right),$$

where I(·;·) is mutual information, and $v≐{\left(v_{1},v_{2}\right)}^{T}$. Here we define

(13) $$\begin{array}{c} R^{-}≐I\left(x;y\right)-I\left(x;v\right),\;\;R^{+}≐I\left(x;y|v\right). \end{array}$$

We will show that the bounds are tight in our model when the total transmitting power of the helpers is large enough, then the upper bound could be a proper substitution of the secret-key capacity to show the performance of the proposed algorithms.

We know

(14) $$\begin{array}{c} I(x;y|v)=H(x|v)-H(x|yv), \end{array}$$

then we have

(15) $$H\left(x|v\right)=\log|Q_{xv}\left|-\log\right|Q_{v}|+\log\left(\pi e\right),$$

(16) $$H\left(x|yv\right)=\log|Q_{xyv}\left|-\log\right|Q_{yv}|+\log\left(\pi e\right),$$

where $Q_{xv}$, $Q_{v}$, $Q_{xyv}$ and $Q_{yv}$ are all covariance matrices, which are given as

$$\begin{array}{ccc} Q_{xv} & ≐ & E\left(\left[\begin{array}{c} x\\ v \end{array}\right]\left[x^{*},v^{H}\right]\right), \end{array}$$

$$\begin{array}{ccc} Q_{v} & ≐ & E(vv^{H}), \end{array}$$

$$\begin{array}{ccc} Q_{xyv} & ≐ & E\left(\left[\begin{array}{c} x\\ y\\ v \end{array}\right]\left[x^{*},y^{*},v^{H}\right]\right),\\ Q_{yv} & ≐ & E\left(\left[\begin{array}{c} y\\ v \end{array}\right]\left[y^{*},v^{H}\right]\right). \end{array}$$

Then we have

(17) $$\begin{array}{ccc} \left|Q_{xv}\right| & = & \;\;\;\;\sum_{i>j>k}\alpha_{ijk}P^{3}+\sigma_{n}^{2}\left(\sum_{i>j}\beta_{ij}P^{2}+\sigma_{n}^{2}\left(\sum_{i}\gamma_{i}P+\sigma_{n}^{2}\right)\right), \end{array}$$

(18) $$\begin{array}{c} \left|Q_{v}\right|=\sum_{i>j}\mu_{ij}P^{2}+\sigma_{n}^{2}\left(\sum_{i=1}^{N}\eta_{i}P+\sigma_{n}^{2}\right), \end{array}$$

(19) $$\begin{array}{c} \left|Q_{yv}\right|=\rho \left|Q_{xv}\right|+\left(1-\rho \right)\sigma_{n}^{2}\left|Q_{v}\right|, \end{array}$$

(20) $$\begin{array}{c} \left|Q_{xyv}\right|=\left(1+\rho \right)\sigma_{n}^{2}\left|Q_{xv}\right|-\rho \sigma_{n}^{4}\left|Q_{v}\right|, \end{array}$$

where

(21) $$\begin{array}{ccc} \alpha_{ijk} & = & \rho {\left|e_{j}h_{i}h_{k}\left(\frac{e_{i}}{g_{i}}-\frac{e_{k}}{g_{k}}\;\right)-e_{i}h_{j}h_{k}\left(\;\frac{e_{j}}{g_{j}}\;\;-\;\;\frac{e_{k}}{g_{k}}\;\right)-e_{k}h_{i}h_{j}\left(\;\frac{e_{i}}{g_{i}}\;\;-\;\;\frac{e_{j}}{g_{j}}\;\right)\right|}^{2}, \end{array}$$

(22) $$\begin{array}{ccc} \beta_{ij} & = & {\left|h_{i}e_{j}-h_{j}e_{i}\right|}^{2}\;+\;\rho {\left|\frac{h_{i}h_{j}e_{j}}{g_{j}}-\frac{h_{j}h_{i}e_{i}}{g_{i}}\right|}^{2}\;+\rho {\left|\frac{e_{i}h_{j}e_{j}}{g_{j}}-\frac{e_{j}h_{i}e_{i}}{g_{i}}\right|}^{2}, \end{array}$$

(23) $$\begin{array}{ccc} \gamma_{i} & = & |h_{i}{|}^{2}+{\left|e_{i}\right|}^{2}+\rho {\left|\frac{e_{i}h_{i}}{g_{i}}\right|}^{2}, \end{array}$$

(24) $$\begin{array}{ccc} \mu_{ij} & = & \rho {\left|e_{i}e_{j}\right|}^{2}{\left|\frac{h_{i}}{g_{i}}-\frac{h_{j}}{g_{j}}\right|}^{2}, \end{array}$$

(25) $$\begin{array}{ccc} \eta_{i} & = & {|e_{i}|}^{2}\left(1+\rho {\left|\frac{h_{i}}{g_{i}}\right|}^{2}\right). \end{array}$$

All the above coefficients are positive, and because

(26) $$\beta_{ij}-\mu_{ij}={\left|h_{i}e_{j}-h_{j}e_{i}\right|}^{2}+\rho {\left|\frac{h_{i}h_{j}e_{j}}{g_{j}}-\frac{h_{j}h_{i}e_{i}}{g_{i}}\right|}^{2}>0,$$

(27) $$\gamma_{i}-\eta_{i}={\left|h_{i}\right|}^{2}>0,$$

the coefficients of $\left|Q_{yv}\right|$ and $\left|Q_{xyv}\right|$ are all positive too.

We also have

(28) $$\begin{array}{ccc} I(x;y)-I(x;v) & = & H(x|v)-H(x|y), \end{array}$$

where

(29) $$\begin{array}{c} H\left(x|y\right)=\log\left(\frac{\left(1+\rho \right)\sigma_{n}^{2}\sum_{i=1}^{N}{\left|h_{i}\right|}^{2}P+\sigma_{n}^{4}}{\rho \sum_{i=1}^{N}{\left|h_{i}\right|}^{2}P+\sigma_{n}^{2}}\right)+\log\left(\pi e\right). \end{array}$$

Then we have the following theorem:

**Theorem** **1.** *When there are at least three nodes sending signals, we have* $\lim_{P\to \infty }\left(R^{+}-R^{-}\right)=0$.

**Proof.**  Please see Appendix A. ☐

The result of Theorem 1 is simple, and can be explained from another point of view: When the signal power is infinite, *x* and *y* are almost the same, then *x*, *y* and v almost form a Markov chain. Consequently, from the conclusion in [7], the bounds are tight.

Theorem 1 limits the number of helpers to be at least three. Because the eavesdropper has two inputs during the first two stages, he can then be considered as a receiver equipped with two antennas. When there are only one or two helpers sending signals, the eavesdropper can decode the signals especially when SNR is high. If we want to achieve positive secret-key rate, there should be at least three helpers who send signals with large enough transmitting power in the system.

From Theorem 1, we know that when the transmitting power is large enough the lower bound and upper bound of the secret-key rate of the proposed scheme are almost the same, that is, the bounds of the secret-key capacity are tight. In the following parts of the paper, we just analyze the upper bound of the secret-key capacity.

If we set the total transmit power of the second stage to be the same as that of the first stage, namely

(30) $$\rho \sum_{i=1}^{N}{\left|\frac{h_{i}}{g_{i}}\right|}^{2}P=NP,$$

then we have $\rho =\frac{N}{\sum_{i=1}^{N}{\left|\frac{h_{i}}{g_{i}}\right|}^{2}}$, when *P* goes to infinite, we have

(31) $$\begin{array}{ll} & \lim_{P\to \infty }R^{+}\\ = & \lim_{P\to \infty }\log NP+\log\left(\frac{\sum_{i>j>k}\alpha_{ijk}}{N\sum_{i>j}\beta_{ij}}\right)-\log\left(1+\frac{\sigma_{n}^{2}}{N}\sum_{i=1}^{N}{\left|\frac{h_{i}}{g_{i}}\right|}^{2}\right). \end{array}$$

Actually, $\sum \alpha_{ijk}$ is impossible to be zero in a practical system. In high SNR region, $R^{+}$ increases linearly with the total power log(P). This means such a simple algorithm can help to get infinite secret-key rate by increasing the total signal power without considering any information of the eavesdropper.

## 4. Threat Model Analysis

The above analysis on secret-key rate is based on the information about the eavesdropper, we can only get a theoretical upper bound of system performance. Actually, in a real system, we have no idea of the potential eavesdropper, so the information-theoretic security is not available. In this section, we discuss the practical challenges on the system without assumptions on the eavesdropper.

### 4.1. Passive Attacks

Since a passive eavesdropper does not send any signal, it is hard to estimate how much information is leaked to such an attacker. Different stage of the process has different level of the possibility to leak the confidential messages.

**ST1-B and ST2-B:** During these sub-stages, the partner A and MC send channel probing signals to the helpers, then they can estimate the channel gains of themselves. For a passive eavesdropper, he can also get the channel probing signals, then the channels state information of his own is known by the eavesdropper. However, the legitimate system links is still unknown by the eavesdropper. In a poor multipath scattering environment, the eavesdropper may have a strong correlation in measurements of the wireless channels. However, since there are many helpers in the system, the eavesdropper could not have correlation with all the helpers. So when the helper number is large enough, the leaking information to the eavesdropper is limited.

**ST1-F and ST2-F:** In these sub-stages, all the helpers send signals simultaneously to partner A. The eavesdropper can also get the signals. However, the two signals are different linear combination of $s_{i}\left(k\right),i=1,\ldots ,N$. When the node number *N* goes to infinite, the eavesdropper’s signals is independent of what node A and MC receive. More node number can help the system to achieve higher security level.

**Figure 4:**
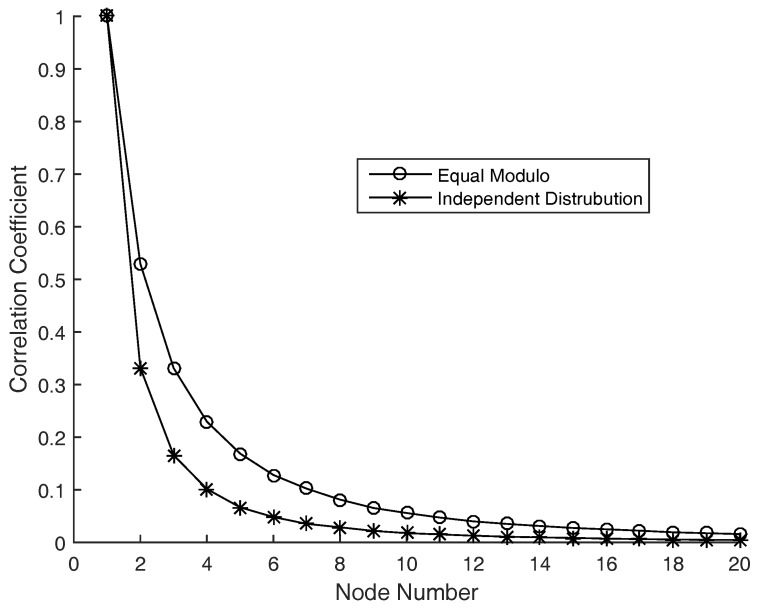
Average correlation coefficients of x(k) and $v_{1}\left(k\right)$ in ST1 vs. helper number. Equal Modulo means $|h_{i}\left|=\right|e_{i}|,i=1,\ldots ,N$, with $h_{i},i=1,\ldots ,N$ are independent Rayleigh fading channels, and $e_{i}=\left|h_{i}\right|e^{j\phi_{i}}$ where $\phi_{i},i=1,\ldots ,N$ are independent random phases uniformly distributed within [0,2π). Independent Distribution means $h_{i}$ is independent of $e_{i}$, with $h_{i},i=1,\ldots ,N$ and $e_{i},i=1,\ldots ,N$ are all independent Rayleigh fading channels. We do independent experiments 10,000 times to get the average values without considering the receiving noises.

When the eavesdropper is placed very near A or MC, or else the whole system works in a poor multipath scattering environment, the eavesdropper’s channels might have a strong correlation with the legitimate users. In these cases, the phase of the complex channel coefficients is usually still independent of each others, while the modulo of the channel coefficients or the RSS (received signal strength) are correlated to each others. The proposed scheme is even very sensitive of the phases, which is demonstrated in Figure 4.

In Figure 4, the worst and best cases are demonstrated. The best situation is that the eavesdropper’s channels are independent of the legitimate users’. The worst case is that the eavesdropper’s channels are highly related with the legitimate users’, even the extreme case is $|h_{i}\left|=\right|e_{i}|$, that is the eavesdropper’s channels have the same modulo of A’s. We consider the average correlation coefficient of *x* and $v_{1}$ to show how the helper number improve the system security. The average correlation coefficient is defined as

(32) $$\zeta =E_{h,e}\left(\frac{\left|Cov(x,v_{1})\right|}{\sqrt{Var\left(x\right)Var\left(v_{1}\right)}}\right).$$

It is shown in Figure 4 that even the channels are highly correlated, the signals intercepted by the passive eavesdropper is almost independent of the legitimate receiver’s when the helper number is large enough, which means the eavesdropper almost could not get any useful information from the receiving signals.

**ST3:** ST3 is usually an error correction process. Due to error of channel measuring and noise, the extracted bits at A and MC sides are usually not identical. During this stage, parity bit information may be transmitted openly to correct errors. The eavesdropper can get the messages in this stage. In order to eliminating the eavesdropper’s partial information about the key, there will be a privacy amplification process [6]. In the privacy amplification phase, both legitimate parts compress the information to their “real entropy”. However, in a practical system, the information leaked to the eavesdropper is hard to estimated, then the “real entropy” is hard to be decided. A possible solution is to get average or maximum leaking information by experiments.

**Multi-Antenna attack:** The most threatening passive attack is multi-antenna attack, that is, the eavesdropper is equipped with multiple antennas to intercept the signals. In this case, the eavesdropper performs like a MIMO (Multiple-In-Multiple-Out) system . In an ideal situation, when the antenna number of the eavesdropper is unlimited, the eavesdropper could know exactly the signals from every transmitting antenna. Then the receiving signals of the eavesdropper can be considered as $s_{i},i=1,\ldots ,N$. It is easy to know that $I(x;y|s_{1},\ldots ,s_{N})=0$, then the secret-key rate of the system is down to zero, that is, the system cannot get information theory secrecy any more. However we will prove in a following subsection that the system is still secure with enough number of helpers.

### 4.2. Active Attacks

In [36], the author classifies the now existing active attacks into three types: disruptive jamming attack, manipulative jamming and channel manipulation attack.

**Disruptive jamming:** The purpose of disruptive jamming attacks is to minimize the key generation rate between legitimate users. The jamming signals can be injects in every stage of the proposed scheme. Most harmful behavior is to disrupt the channel probing process, without accurate channel estimation, the secret-key rate of the system will be dramatically deduced. A possible solution of this issue is proposed in [36], random probing signals is used to hide the channel state information, which is also suitable of the scheme of this paper.

**Manipulative jamming:** In [37], a manipulative attack is proposed (Man-In-The-Middle Attack) to control the channel measurements at legitimate users. In our proposal, it does not work, any misleading of the channel measurements will cause failure on the key generation. Manipulative jamming on channel probing process will deduce the secret-key rate instead of compromising the generated key.

A possible way of manipulative jamming attack is that the attacker can transmit signals with high enough power in substage ST1-F and ST2-F, then the receiving signals of A and MC are mainly controlled by the attacker, thus the generated key is compromised by the malicious third-party. Since the attacker acts just like a normal helper, this type of attack is hard to be defended against. One possible solution to address the issue is power detecting: After generating a new key, all the helpers can report through the open channel about the average power during the period they transmitted to MC (This action will leak part of the channel information to the eavesdropper, which should be considered in ST3). Then MC can compare the average power that he has received and the messages helpers reports. If there be an attacker, the receiving signal power will be higher than the sum of all the reported power, which means the generated key is possibly manipulated. The more attacker controls, the more easy he would be detected.

**Channel manipulation attack:** Because the key is not generated from the channel information, channel manipulation attack can not influence the key generation process of this paper. On the other hand, since the system is equipped in a train which travels through a long distance, channel manipulation is almost impossible for any potential attacker.

### 4.3. Multi-Antenna Attack

From the scheme, we know that the eavesdropper cannot get any information of the channel gain $h_{i},i=1,2,\ldots ,N$ from his receiving signals $v_{1}$ and $v_{2}$. When information theory secrecy is not achievable, the unknown of $h_{i}$ can still help the system to achieve computationally secure secrecy.

The term of secret-key rate is based on information theory secrecy, or unconditionally secure secrecy. When the eavesdropper is equipped with multiple antennas, or else there are multiple cooperative eavesdroppers, the performance of the system will be lower. If number of the antennas or the eavesdroppers is infinite, the eavesdropper could possible almost know what the helpers send. In this case the system cannot achieve unconditional secrecy any more. However, the system is still computationally secure, which means cracking the secret-key is equivalent to the solution of some problem known to be laborious.

The analysis of this case is valuable, because in a practical system, we cannot limit the number of the eavesdropper’s antenna. The proposed scheme is computationally secure when there are infinite eavesdroppers. The reason is that in our system what the helpers send is different from what the users receive, multiple antennas can help the eavesdropper to get the sending signals but not the receiving signals. Without knowledge of the legitimate user’s channels, the eavesdropper still cannot crack the secret-key.

We assume that when there are infinite eavesdroppers, they could know exactly what the helpers send during the first two stages in an ideal situation. The eavesdroppers know the symbols $s_{i},i\;=\;1,2,\ldots ,N$ and $\sqrt{\rho }\frac{h_{i}}{g_{i}}$, *i* =1, ..., *N*, but they have no idea about $h_{i},i=1,2,\ldots ,N$. The method for the eavesdroppers to crack the secret-key is just guess. Then the problem is what is the probability for the eavesdropper to crack the key for one trial.

Since the receiving signals of the two legitimate users and the eavesdropper are all Gaussian signals, we consider

(33) $$s=\sum_{i=1}^{N}h_{i}s_{i},$$

as the effective signals, then user A, B and the eavesdropper all get a noisy version of *s*. We re-model the signals at the legitimate users as

(34) $$x=s+n_{1},\;y=\sqrt{\rho }s+n_{2}.$$

When the eavesdropper tries to estimate $h_{i},i=1,\ldots ,N$ with $\hat{h}$, where $\hat{h}≐{\left({\hat{h}}_{1},{\hat{h}}_{2},\ldots ,{\hat{h}}_{N}\right)}^{T}$ denotes random selected complex vector as the estimation of the CSI, then the estimated signals can be written as

(35) $$\hat{v}=\sum_{i=1}^{N}{\hat{h}}_{i}s_{i}=\delta s+n_{e},$$

where $n_{e}$ denotes the equivalent noise of the estimation and is independent of *s*, then we have

$$\begin{array}{ccc} \;\;\;\;\delta & = & \frac{\sum_{i=1}^{N}h_{i}^{*}{\hat{h}}_{i}}{\sum_{i=1}^{N}{\left|h_{i}\right|}^{2}},\\ \;\;\;\;E(|n_{e}{|}^{2}) & = & P\frac{\sum_{i=1}^{N}|h_{i}{|}^{2}\sum_{i=1}^{N}{\left|{\hat{h}}_{i}\right|}^{2}-{\left|\sum_{i=1}^{N}h_{i}{\hat{h}}_{i}^{*}\right|}^{2}}{\sum_{i=1}^{N}{\left|h_{i}\right|}^{2}}≐\sigma_{e}^{2}. \end{array}$$

The SNRs of the legitimate users and the eavesdropper are

(36) $$\begin{array}{ccc} \eta_{1} & = & \frac{P}{\sigma_{n}^{2}}\sum_{i=1}^{N}{\left|h_{i}\right|}^{2},\eta_{2}=\frac{NP\sum_{i=1}^{N}{\left|h_{i}\right|}^{2}}{\sigma_{n}^{2}\sum_{i=1}^{N}{\left|\frac{h_{i}}{g_{i}}\right|}^{2}}, \end{array}$$

(37) $$\begin{array}{ccc} \eta_{e} & = & \frac{{\left|\sum_{i=1}^{N}h_{i}{\hat{h}}_{i}^{*}\right|}^{2}}{\sum_{i=1}^{N}|h_{i}{|}^{2}\sum_{i=1}^{N}{\left|\hat{h_{i}}\right|}^{2}-{\left|\sum_{i=1}^{N}h_{i}{\hat{h}}_{i}^{*}\right|}^{2}}. \end{array}$$

If we consider the trial of the eavesdropper as an observation of the random source *s*, we can compute a secret-key rate of the system, ${\hat{R}}_{k}$, where $I\left(x;y\right)-I\left(x;\hat{v}\right)\leq {\hat{R}}_{k}\leq I\left(x;y|\hat{v}\right)$. When the legitimate users want to generate secret-key with rate $R_{0}$, any secret-key generation process of our model with $R_{0}$ lower than ${\hat{R}}_{k}$ could be secure. In addition, if the secret-key generating rate is higher than ${\hat{R}}_{k}$, the system is no longer secure, or else, we can say in this case the eavesdropper can crack the key. Then we have the following theorem:

**Theorem** **2.** *If the system channel gain* h *is statistically independent complex Gaussian random variables with the same variance* $\sigma^{2}$, $\hat{h}$ *is the channel estimation vector, the legitimate users tend to generate secret-key with rate* $R_{0}$*, the average probability of one trial for the eavesdroppers to crack the key is*

(38) $$\begin{array}{cc} & E\left(Pr\left\{{\hat{R}}_{k}<R_{0}\right\}\right)\\ = & {\left(\sqrt{{\left(\frac{\eta_{1}+\eta_{2}}{2}\right)}^{2}+\frac{\eta_{1}\eta_{2}}{e^{R_{0}}-1}}-\left(\frac{\eta_{1}+\eta_{2}}{2}\right)\right)}^{1-N}. \end{array}$$

**Proof.**  Please see Appendix B. ☐

Theorem 2 means when we want to achieve secret-key rate of $R_{0}$, if the SNRs at the legitimate users, $\eta_{1}$ or $\eta_{2}$, are large enough, and the number of the helpers is also large enough, the probability for the eavesdropper to crack the key can be arbitrary small.

For example, when $\eta_{1}$ and $\eta_{2}$ are both 20 dB and we want to achieve the secret-key rate of 1 nats/symbol, and there are 20 helpers. Then the probability is about $10^{-27}$, this means if the eavesdropper wants to ensure 90% probability to get the proper secret-key, he have to do about $2\times 10^{27}$ independent trials, which is almost impossible to be done.

Note that Theorem 2 does not limit the probability density function of $\hat{h}$, which means the eavesdropper can guess the channel gains in any way as he will, and the this will not affect the average probability for him to get the secret-key.

## 5. Numerical Results

In this section, we demonstrate the performance of the proposed schemes numerically. We perform the simulations with three types of configure: fixed channels, Rayleigh fading channels and line-of-sight (LOS) channels. In this section, all the notion of secret-key rate is actually the upper bound of the secret-key capacity.

**Figure 5:**
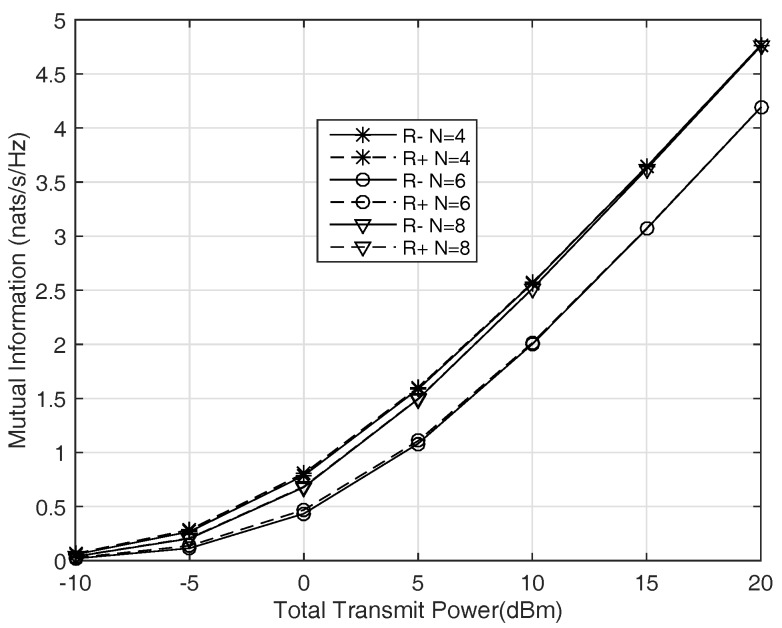
Upper and lower bound of secret-key capacity with the channels listed in Table 1. In the figure, $R^{+}$ and $R^{-}$ are close to each other, which verifies the result of Theorem 1.

We randomly generate some channel gains shown in Table 1, the noise power $\sigma_{n}^{2}$ is 0 dBm. We compare the $R^{-}$ and $R^{+}$ in Figure 5 . We can see that $R^{-}$ and $R^{+}$ are very close to each other, especially when the transmitting power is high, they are almost the same. Figure 5 shows that $R^{+}$ is a tight upper bound of the secret-key capacity, and verifies the result of Theorem 1.

**Figure 6:**
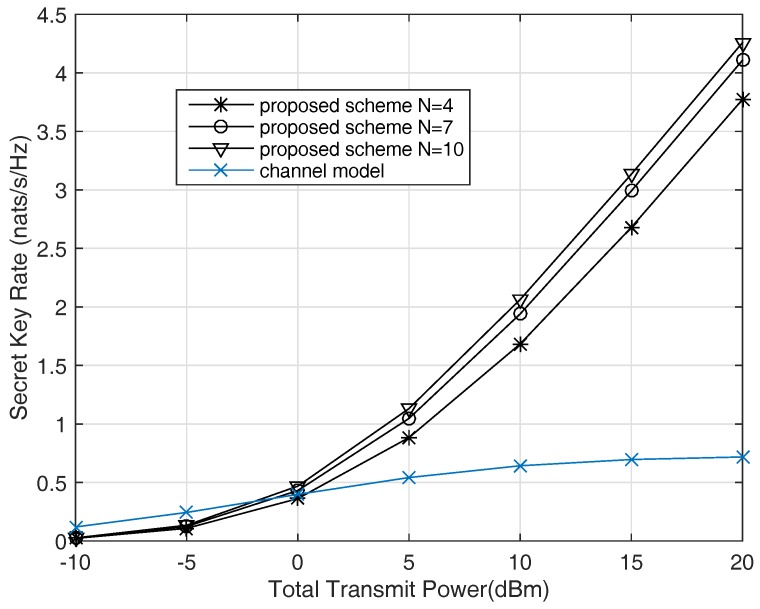
Average secret-key rate versus transmitting power for Rayleigh fading channels.

In Figure 6 we consider wireless communication system in fading environment. The channels of the users and the eavesdropper are all Rayleigh fading channels which are independent of each other, and follow the unit variance zero mean complex Gaussian distribution. The noise power $\sigma_{n}^{2}$ is also set as 0 dBm. We compare the results with different numbers of the helpers and different algorithms, 1000 times of experiments are performed to get average secret-key rate, which are shown in Figure 6. The secret-key rates increase linearly with the total transmitting power, as shown in (31), and more helpers result in better performance. Even when we have no idea about the eavesdropper, the performance of the algorithm is fairly good.

Figure 6 also shows the performance of a simple key generation scheme of channel model (Section 4.1 in [35]). In this model, the common randomness is from user A, that is, user A sends random signals to MC, and then the two users try to generate a secret-key from these random signals by public discussion. This is a typical channel model for secret-key generation. We implement the same transmitting power configure as the proposed schemes. It is shown in Figure 6 that the performance of channel model saturates with the increasing of the transmitting power, the secret-key rate is much lower than the proposed schemes in high SNR region.

Secret-key generation process is not a common communication process, whose transmitting rate increases with the transmitting power. Consider the lower bound of secret-key capacity, $R^{-}=I\left(x;y\right)-I\left(x;v\right)$. The first part of $R^{-}$ is the mutual information of *x* and *y*, which increases with the transmitting power. In addition, the second part of $R^{-}$ will also increase with the transmitting power. This means when antenna power increases, the mutual information of *x* and *y* is larger, and the leaked information to the eavesdropper is also larger. Then if the correlated random source observed by the two legitimate parties is some types of radio signals, higher sending power would not benefit the system performance much. However, the proposal in this paper performs just like a common communication system. The reason is that the leaked information to the eavesdropper is almost fixed, which is mainly determined by the correlation coefficient of *x*, *y* and v. The eavesdropper does not get more information when the signal power is higher, that is, I(x;v) does not increase with the signal power.

**Figure 7:**
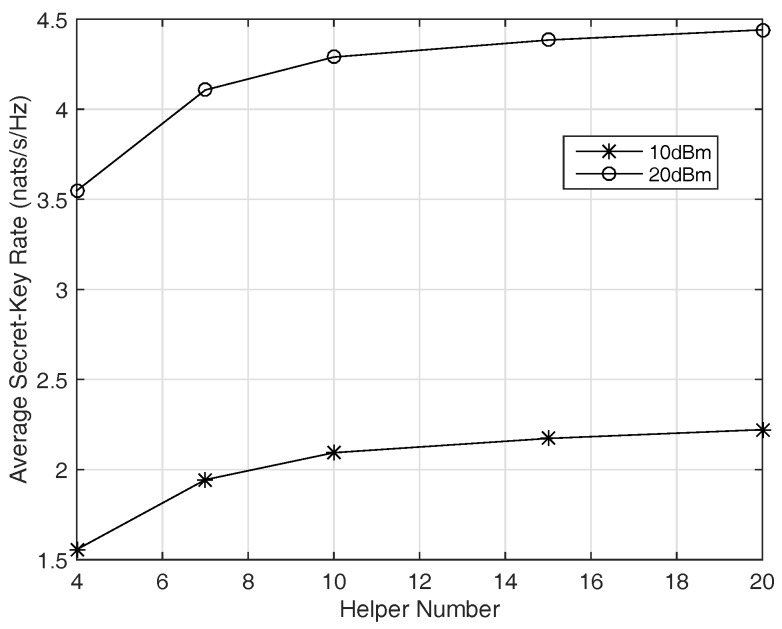
Average secret-key rate versus helper number for Rayleigh fading channels with total transmitting power of 10 dBm and 20 dBm.

Figure 7 illustrates the secret-key rate of the system versus helper number with the same configure as in Figure 6. It is shown that more helpers result in better performance in average, but the secret-key rate increases more slowly when the helper number becomes large. The reason is when the number of helpers is large enough, the receiving signals of the eavesdropper are almost independent of the legitimate users’ (as shown in Figure 4), the secret-key rate is almost saturated to its upper bound I(x;y). Then more helpers benefit a little to the system performance when the helper number is large. In a practical system, more helpers cause higher system complexity, then there would be a trade-off between system complexity and secret-key rate.

**Figure 8:**
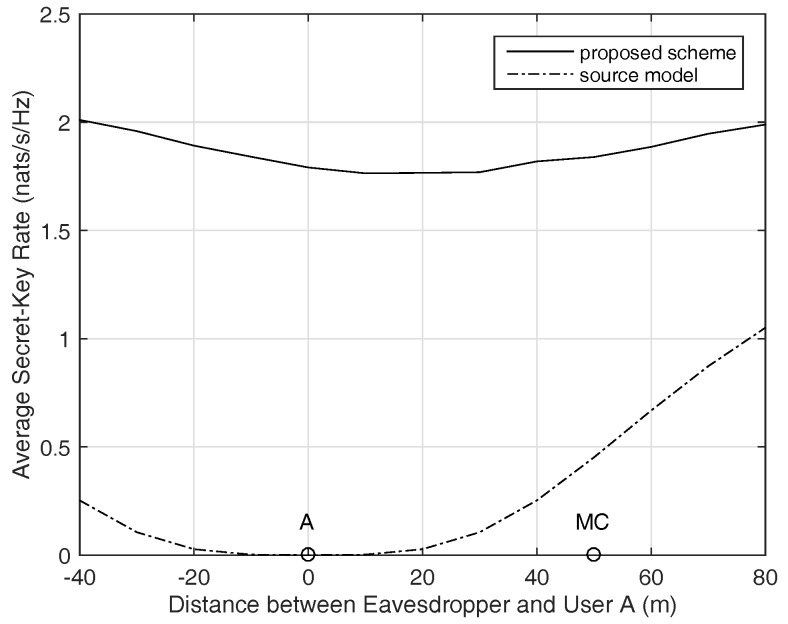
Average secret-key rate of LOS channel when the eavesdropper moves along the horizonal line.

Figure 8 compares the proposed schemes and the channel model key generation scheme in LOS channel. In the experiment, the eavesdropper moves along the horizonal line between A and MC. Channels between any two nodes are modeled by a simple line-of-sight channel model including the path loss effect and a random phase: $h=d^{-c/2}e^{j\theta }$ where *d* is the distance between any two nodes, c=3.5 is the path loss exponent, θ is the random phase uniformly distributed within [0,2π). There are four helpers placed along the horizonal line, with equal spacing of 20 m. The noise power is $\sigma_{n}^{2}$ = −60 dBm. It is shown that the secret-key rate of channel model is almost zero when the eavesdropper is placed close to user A, while the proposed schemes model can still achieve fairly good performance near the two users. In this case, the channels of the system are highly correlated to each other, most of the physical layer secret-key generation schemes could not achieve fairly good performance except for the algorithm in this paper. This is ascribed to the sensitivity of the proposal.

We do not compare the proposed schemes with other CSI based key generation algorithms of [13,14,15,16,17,18,19,20,21,22,23,24,25,26,27,28,29,30,31,32,33] in numerical simulations, because the system configures are different. All the CSI based algorithms depend on the coherence time and bandwidth of the wireless channel models, while the performance our scheme mainly depends on the symbol rate of the helpers. Typically, coherent time of a wireless communication system would be longer than 10 milliseconds, then for a narrow band system, there will be up to several thousands of bits secret-key generated by the system per second in high SNR region with small number of relays. In addition, for the scheme in this paper, hundreds of thousands of bits secret-key could be generated per second with only 10 kHz bandwidth in high SNR region.

## 6. Conclusions

In this paper, we have investigated the design of the correlated Gaussian sources with multiple cooperative helpers for physical layer secret-key generation for TSSN. The proposed scheme can help to update the secret-key of the wireless sensors in the system dynamically and securely.

In traditional distributed physical layer security communication systems, cooperative helpers are used as relays or interference sources. The basic idea of these algorithms is beam-forming. The main difference between the proposed schemes and the traditional algorithms is that the helpers send independent random signals in our schemes. What the helpers send is different from what the users receive, even the helpers themselves have no idea of the receiving signals of the users. This helps to create spatial differences between the legitimate users and the eavesdropper.

The proposed scheme provides an artificial random source for secret-key generation, then it is possible to get high secret-key rate by increasing the symbol rate of the helpers. Traditional CSI based secret-key generation schemes can only achieve up to several hundreds of bits secret-key per second for narrow band system, and highly depend on the coherence time of the channels. The proposed scheme, by contrast, can achieve hundreds of thousands of bits per second for narrow band system, and could possibly generate several mega bits secret-key per second for wideband system.

Note that when there are too many helpers, the synchronization is difficult for a practical system. The estimation of the channel CSI could not be accurate, the estimation errors accumulate with the increasing of the helper number. These facts will destroy the correlationship of the legitimate users, and suffer the performance of the system. How to find a balance or an optimal solution for a practical system with these issues is left for future work.

## Figures and Tables

**Table 1:** CSI list for simulation.

Number	Channel Gain
N = 4	$h=\left\{0.045+1.645i;2.416-0.454i;-0.309+1.008i;0.187+2.049i\right\}$
	$g=\left\{0.531+1.371i;2.134+0.245i;0.354+0.118i;0.231+0.384i\right\}$
	$e=\left\{0.807+0.637i;-1.027-0.404i;1.294-0.403i;0.014+0.084i\right\}$
N = 6	h={0.947+0.602i;−0.525+0.017i;−1.115−1.610i;−1.592+1.238i;1.174+0.683i; 0.485−0.780i}
	g={1.288−0.070i;−0.013−0.578i;−1.333+0.469i;−0.556+1.299i;0.755+1.634i; −0.911−0.702i}
	e={0.218−0.435i;1.713−0.562i;−2.078+0.878i;0.112−0.814i;−1.086−0.258i; −1.558+0.493i}
N = 8	h={−0.236−1.674i;−0.105−0.768i;2.682−1.610i;−1.530−0.405i;−0.001−0.530i; 0.773+0.315i;0.458+1.547i;−0.331+0.611i}
	g={−1.213−0.691i;−0.274+0.767i;−1.414−1.255i;−0.713−0.016i;1.081−1.448i; −0.291+0.249i;1.597+0.715i;0.085−1.031i}
	e={0.579+0.484i;−0.642+0.787i;−0.211+0.426i;−1.332−0.176i;−0.868−0.136i; −1.469−0.955i;−1.562−0.097i;0.127+0.407i}

## Appendix A. Proof of Theorem 1

**Proof.** Because $|h_{i}{|}^{2}>0,i=1,2,\ldots ,N$

(A1) $$\begin{array}{cc} & \lim_{P\to \infty }H\left(x|y\right)\\ = & \lim_{P\to \infty }\log\left(\frac{\left(1+\rho \right)\sigma_{n}^{2}\sum_{i=1}^{N}{\left|h_{i}\right|}^{2}P+\sigma_{n}^{4}}{\rho \sum_{i=1}^{N}{\left|h_{i}\right|}^{2}P+\sigma_{n}^{2}}\right)+\log\left(\pi e\right)\\ = & \log\left(\left(1+1/\rho \right)\sigma_{n}^{2}\right)+\log\left(\pi e\right), \end{array}$$

then from Equations (17) and (18) we have

(A2) $$\begin{array}{cc} & \lim_{P\to \infty }|Q_{v}\left|/\right|Q_{xv}|\\ = & \lim_{P\to \infty }\;\frac{\sum \mu_{ij}P^{2}+\sigma_{n}^{2}\sum \eta_{i}P+\sigma_{n}^{4}}{\sum \alpha_{ijk}P^{3}\;+\;\sigma_{n}^{2}\;\sum \;\beta_{ij}P^{2}\;+\;\sigma_{n}^{4}\;\sum \;\gamma_{i}P\;+\;\sigma_{n}^{6}} \end{array}$$

There are at least three helpers sending signals, which means $\sum \alpha_{ijk}>0$, then we have

(A3) $$\lim_{P\to \infty }|Q_{v}\left|/\right|Q_{xv}|=0,$$

then

(A4) $$\begin{array}{cc} & \lim_{P\to \infty }H\left(x|yv\right)\\ = & \lim_{P\to \infty }\log\left(\frac{\left(1+\rho \right)\sigma_{n}^{2}\left|Q_{xv}\right|-\rho \sigma_{n}^{4}\left|Q_{v}\right|}{\rho \left|Q_{xv}\right|+\left(1-\rho \right)\sigma_{n}^{2}\left|Q_{v}\right|}\right)+\log\left(\pi e\right)\\ = & \log\left(\left(1+1/\rho \right)\sigma_{n}^{2}\right)+\log\left(\pi e\right). \end{array}$$

Finally from Equations (14) and (28) we know

(A5) $$\begin{array}{c} \lim_{P\to \infty }\left(R^{+}-R^{-}\right)=\lim_{P\to \infty }\left(H(x|yv)-H(x|y)\right)=0. \end{array}$$

 ☐

## Appendix B. Proof of Theorem 2

**Proof.** Assuming that the channel estimation vector $\hat{h}$ follows the probability density function of $L(\hat{h})$. Because $C_{sk}\leq I\left(x;y|\hat{v}\right)$, for the proposed algorithm we have

(A6) $$\begin{array}{cc} R^{+} & =I(x;y|\hat{v})\\ & =H\left(x|\hat{v}\right)-H\left(x|y\hat{v}\right)\\ & =\log\frac{|Q_{x\hat{v}}\left|\right|Q_{y\hat{v}}|}{|Q_{\hat{v}}\left|\right|Q_{xy\hat{v}}|}. \end{array}$$

We assume that the power of signal s(k) is $P_{s}$, that is,

(A7) $$P_{s}≐E\left(ss^{*}\right).$$

From Equations (34) and (35) we have

(A8) $$\eta_{1}=\frac{P_{s}}{\sigma_{n}^{2}},\;\eta_{1}=\frac{\rho P_{s}}{\sigma_{n}^{2}},\;\eta_{e}=\frac{{\left|\delta \right|}^{2}P_{s}}{\sigma_{e}^{2}}.$$

Then we have

(A9) $$\begin{array}{cc} |Q_{x\hat{v}}| & =\left|\begin{array}{cc} P_{s}+\sigma_{n}^{2}, & \delta^{*}P_{s}\\ \delta^{*}P_{s}, & {\left|\delta \right|}^{2}P_{s}+\sigma_{e}^{2} \end{array}\right|\\ & ={\left|\delta \right|}^{2}P_{s}\sigma_{n}^{2}+P_{s}\sigma_{e}^{2}+\sigma_{n}^{2}\sigma_{e}^{2}\\ & =\sigma_{n}^{2}\sigma_{e}^{2}\left(\eta_{1}+\eta_{e}+1\right). \end{array}$$

Following almost the same steps, we have

(A10) $$\begin{array}{ccc} |Q_{y\hat{v}}| & = & \sigma_{n}^{2}\sigma_{e}^{2}\left(\eta_{2}+\eta_{e}+1\right), \end{array}$$

(A11) $$\begin{array}{ccc} |Q_{xy\hat{v}}| & = & \sigma_{n}^{4}\sigma_{e}^{2}\left(\eta_{1}+\eta_{2}+\eta_{e}+1\right), \end{array}$$

(A12) $$\begin{array}{ccc} |Q_{\hat{v}}| & = & \sigma_{e}^{2}\left(\eta_{e}+1\right). \end{array}$$

Then we have

(A13) $$R^{+}=I\left(x;y|\hat{v}\right)=\log\frac{\left(\eta_{1}+\eta_{e}+1\right)\left(\eta_{2}+\eta_{e}+1\right)}{\left(\eta_{e}+1\right)\left(\eta_{1}+\eta_{2}+\eta_{e}+1\right)}.$$

Actually, $R^{+}$ is a decreasing function of $\eta_{e}$. Because $\eta_{e}>0$, then for given $R^{+}=R_{0}$, $\eta_{1}$ and $\eta_{2}$, if

(A14) $$\eta_{e}>\sqrt{{\left(\frac{\eta_{1}+\eta_{2}}{2}\right)}^{2}+\frac{\eta_{1}\eta_{2}}{e^{R_{0}}-1}}-\left(\frac{\eta_{1}+\eta_{2}}{2}\right)-1≐\eta_{0},$$

We have ${\hat{R}}_{k}\leq R_{0}$. Then from Equation (37) we have

(A15) $$\frac{{\left|\sum_{i=1}^{N}h_{i}{\hat{h}}_{i}^{*}\right|}^{2}}{\sum_{i=1}^{N}|h_{i}{|}^{2}\sum_{i=1}^{N}{\left|\hat{h_{i}}\right|}^{2}}>\frac{\eta_{0}}{1+\eta_{0}}.$$

To compute the probability of the trial, we convert all the complex numbers to real numbers. We define the real channel gain vector and the channel gain estimation vector as

(A16) $$x≐{\left(h_{1}^{\left(r\right)},h_{1}^{\left(i\right)},\ldots ,h_{N}^{\left(r\right)},h_{N}^{\left(i\right)}\right)}^{T},\hat{x}≐{\left({\hat{h}}_{1}^{\left(r\right)},{\hat{h}}_{1}^{\left(i\right)},\ldots ,{\hat{h}}_{N}^{\left(r\right)},{\hat{h}}_{N}^{\left(i\right)}\right)}^{T},$$

where $h_{i}=h_{i}^{\left(r\right)}+jh_{i}^{\left(i\right)},\;{\hat{h}}_{i}={\hat{h}}_{i}^{\left(r\right)}+j{\hat{h}}_{i}^{\left(i\right)},\;i=1\ldots N$. Then the condition (A15) is

(A17) $$\frac{{\left(x^{T}\hat{x}\right)}^{2}+{\left(x^{T}R_{0}\hat{x}\right)}^{2}}{∥x∥∥\hat{x}∥}>\frac{\eta_{0}}{1+\eta_{0}}$$

where

(A18) $$\begin{array}{c} R_{0}≐\left(\begin{array}{ccccc} 0, & -1, & \ldots & 0, & 0\\ 1, & 0, & \ldots & 0, & 0\\ \vdots & & \ddots & \vdots \\ 0, & 0, & \ldots & 0, & -1\\ 0, & 0, & \ldots & 1, & 0 \end{array}\right) \end{array}$$

It is clear that for any vector $x\in R^{2N}$, we have $x^{T}R_{0}x=0$. The probability of the eavesdropper successing to have a estimation of certain h is

(A19) $$\begin{array}{ll} P(x) & =Pr\left\{\frac{{\left(x^{T}\hat{x}\right)}^{2}+{\left(x^{T}R_{0}\hat{x}\right)}^{2}}{∥x∥∥\hat{x}∥}>\frac{\eta_{0}}{1+\eta_{0}}\right\}\\ & =\underset{y}{\underset{︸}{\int \ldots \int }}L\left(y\right)J\left(y,x\right)dy, \end{array}$$

where

(A20) $$J\left(y,x\right)≐\left\{\begin{array}{cc} 1, & \frac{{\left(x^{T}y\right)}^{2}+{\left(x^{T}R_{0}y\right)}^{2}}{∥x∥∥y∥}>\frac{\eta_{0}}{1+\eta_{0}}\\ 0, & others \end{array}\right..$$

Then the average probability of one test to crack the key is

(A21) $$\begin{array}{c} E\left(P(x)\right)=\underset{y}{\underset{︸}{\int \ldots \int }}L\left(y\right)\left(\underset{x}{\underset{︸}{\int \ldots \int }}J\left(y,x\right)N\left(x\right)dx\right)dy, \end{array}$$

where $N\left(x\right)≐\frac{1}{{\left(2\pi \sigma^{2}\right)}^{N}}\exp\left(-\frac{1}{2\sigma^{2}}\sum_{i=1}^{N}\left({h_{i}^{\left(r\right)}}^{2}+{h_{i}^{\left(i\right)}}^{2}\right)\right)$. Then we have

(A22) $$\underset{x}{\underset{︸}{\int \ldots \int }}J\left(y,x\right)N\left(x\right)dx\;=\underset{\begin{array}{c} {\left(u^{T}\hat{u}\right)}^{2}+{\left(u^{T}R_{0}\hat{u}\right)}^{2}>\frac{\eta_{0}}{1+\eta_{0}}\\ ∥u∥=1 \end{array}}{\underset{︸}{\int \ldots \int }}\int_{0}^{\infty }\frac{\exp\left(-\frac{r^{2}}{2\sigma^{2}}\right)}{{\left(2\pi \sigma^{2}\right)}^{N}}r^{2N-1}drdu,$$

where Equation (A22) is derived by transforming the axis with

(A23) $$\begin{array}{c} r=\sqrt{∥x∥},\;u=\frac{x}{\sqrt{∥x∥}},\;\hat{u}=\frac{y}{\sqrt{∥y∥}}. \end{array}$$

We rotate the axis with a matrix A, which satisfies $A\hat{u}=l_{0}$, $AR_{0}A^{T}=R_{0}^{T}$, $A^{T}A=I_{2N}$, where $l_{0}≐{\left(1,0,\ldots ,0\right)}^{T}$. Then we have $u^{T}\hat{u}=v_{1}$, $u^{T}R_{0}\hat{u}=-v_{2}$. Because $\int_{0}^{\infty }\frac{1}{{\left(2\pi \sigma^{2}\right)}^{N}}\exp\left(-\frac{r^{2}}{2\sigma^{2}}\right)r^{2N-1}\;dr=\frac{(N-1)!}{2\pi^{N}}$ , we have

(A24) $$\begin{array}{c} \left(\text{A}22\right)=\underset{\begin{array}{c} v_{1}^{2}+v_{2}^{2}>\frac{\eta_{0}}{1+\eta_{0}}\\ ∥v∥=1 \end{array}}{\underset{︸}{\int \ldots \int }}\frac{(N-1)!}{2\pi^{N}}\left|A\right|dv=\frac{1}{{\left(1+\eta_{0}\right)}^{N-1}}. \end{array}$$

Since ${\underset{︸}{\int \ldots \int }}_{y}L\left(y\right)dy=1$, then finally we have $E\left(P(x)\right)=\frac{1}{{\left(1+\eta_{0}\right)}^{N-1}}$. ☐
